# The impact of pain and nocturnal cramps on sleep quality in Charcot Marie Tooth disease: a case-control study

**DOI:** 10.5935/1984-0063.20210025

**Published:** 2022

**Authors:** Cynthia Coelho Souza, Julia Ribeiro da Silva Vallim, Eduardo Luis de Aquino Neves, Paula Santos Nunes, Iandra Maria Pinheiro de França Costa, Lidiane Carine Lima Santos Barreto, Catarina Andrade Garcez, Adriano Antunes de Souza Araujo

**Affiliations:** 1 Universidade Federal de Sergipe, Departament of Pharmacy - Aracaju - Sergipe - Brazil.; 2 Universidade Federal de São Paulo, Departament of Psychobiology - São Paulo - São Paulo - Brazil.

**Keywords:** Chronic Pain, Nervous System, Polyneuropathies, Sleep, Actigraphy, Charcot-Marie-Tooth disease

## Abstract

**Introduction:**

Charcot-Marie-Tooth disease is an inherited neuropathy that presents two main forms - type 1 and type 2 -, differentiated by the speed of the nervous conduction. Our goal was to assess sleep in Charcot-Marie-Tooth disease and its relationship with pain perception and nocturnal cramps.

**Material and Methods:**

This was a case-control study. The case group was composed of 10 volunteers diagnosed with the type 1 and 23 with the type 2. The control group was composed of 22 individuals from the same family matched by age and gender. Volunteers underwent clinical screening to assess the presence of nocturnal cramps and filled the brief pain inventory, the Chalder fatigue scale, the Epworth sleepiness scale, and the Pittsburgh sleep quality index. Sleep was evaluated by actigraphy.

**Results:**

Type 2 patients presented a more severe perception of pain and fatigue, more time spend awake after sleep onset, and had lower sleep efficiency. The individuals who reported nocturnal cramps also had worse perception of pain, reduced sleep latency, and increased sleep fragmentation.

**Conclusion:**

The Charcot-Marie-Tooth type 2 was related with worse sleep quality, perception of pain, and fatigue and these parameters were negatively related.

## INTRODUCTION

Charcot-Marie-Tooth disease (CMT) is an inherited neuropathy that is classified as type 1, also known as demyelinating form, and type 2, known as axonal form. The first one is caused by a myelin sheath deterioration that results in a reduction in the conduction of the nerve impulse. The second one is a primary axonopathy, and the speed of nerve conduction may be within normal limits or moderately reduced^1-4^.

Despite the difference in nerve impulse conduction, type 2 disease has greater variability in the age of symptoms onset and the disease severity. However, the study by Neves and Kok (2011)^6^ showed that in this multigenerational family evaluated in this study, type 2 had a more severe manifestation of the disease (higher prevalence of Babinski signal), which justifies an investigation of sleep in both subtypes.

The prevalence of Charcot-Marie-Tooth disease (CMT) is one of the highest among hereditary neuromuscular diseases, even surpassing progressive muscular dystrophy of the type of Duchenne, but it varies by country - range 16 to 84%. Symptom’s manifestation begins between the first and second decade of life by first compromising the lower limbs and then slowly progresses to proximal segments. Although the phenotype is similar among members of the same family, the progression of the disease may present individual variabilities^5-7,37^.

Chronic pain is a common complaint in patients diagnosed with the disease, with a prevalence ranging from 23 to 100%. In some cases, this symptom is also accompanied by nocturnal cramps that have an impact on sleep, ability to do exercises, and overall quality of life^8-10^.

Sleep and chronic pain are bi-directionally related: adequate sleep improves physical/psychological symptoms of pain and chronic pain is associated with worse sleep quality^11,12^. Therefore, investigating sleep in Charcot-Marie-Tooth’s disease can be an effective tool to modulate pain and improve the quality of life of patients.

Besides, we demonstrated that patients with CMT type 2 present higher fragmentation of sleep^9^, but there is no evidence so far about the relationship of these modifications with pain perception and nocturnal cramps.

Given these evidences, we hypothesized that the same sleep changes would be observed in our population (expanding to CMT type 1 as well) and that somehow pain perception and nocturnal cramps could be mediating factors. So, the objective of this study was to investigate a possible negative relation between sleep, pain, and nocturnal cramps in a multigenerational family with CMT disease in the state of Sergipe, Brazil.

## MATERIAL AND METHODS

A cross-sectional, descriptive, case-control study was carried out between October and December 2017.

**Standard protocol approvals, registrations, and patient consents:** recruitment was carried out by public health care staff from the cities of Pedrinhas, Cristinápolis, and Tobias Barreto in the state of Sergipe, Brazil. All procedures were approved by the health department of the municipalities and only started after the volunteers signed the informed consent form (CAAE No. 69490117.5.0000.5546, report No. 2.256.850). Underage participants have signed the consent form and their guardians, the informed consent form.

**Inclusion criteria:** diagnosis of CMT, aged between 16 and 65 years. The control group was matched by age and sex and composed of individuals from the same family, without signs and symptoms suggestive of CMT. The diagnosis of CMT disease was made by clinical and electrophysiological evaluation. The distinction in type 1 or 2 was made by considering the conduction speed of the median nerve. Results above 38m/s were considered as type 2 (CMT2) and below, type 1 (CMT1)^13^.

**Exclusion criteria:** a medical history of acute or chronic lung disease, cognitive and/or psychiatric disorders, severe or poorly controlled hypertension, heart failure, chronic kidney disease, severe systemic disease, diabetes, obesity, leprosy, pregnant/lactating women, active smokers, drug users, and cases of refusal of consent.

### Questionnaires

*C***harcot-Marie-Tooth neuropathy score (CMTNS):** the scale applied for the evaluation of the disease severity composed by assessment of sensitive and motor symptoms in lower and upper limbs; by sensitive and motor changes in lower and upper limbs observed during the physical examination and changes in neuro-conduction of sensory and motor nerves obtained during electroneuromyography. Each change in these areas is assigned a maximum score of 36 points. Results below 10 were considered as mild, between 11 and 20 points as moderate and above 21 as severe^14^.

**Brief pain inventory (BPI):** a multidimensional self-assessment tool to assess persistent pain. Consisting of 15 items that assess the existence, severity, location, functional interference, therapeutic strategies applied, and treatment effectiveness^15^. We applied the version validated for Portuguese-BR^16^.

**Chalder fatigue scale (CFS):** a self-administered questionnaire that assesses the extent and severity of fatigue. Composed of 11 items on a scale from 0 to 4 ranging from asymptomatic to maximum symptoms - Likert scoring method^17^. We applied the version validated for Portuguese-BR^18^.

**Epworth sleepiness scale (ESS):** composed of 8 items in which the individual must respond to the probability of napping in each of the situations presented^19^. The score was calculated as described by the version validated for Portuguese-BR^20^.

**Pittsburgh sleep quality index (PSQI):** composed of 19 self-managed questions that are grouped into 7 components, whose scores are added together to produce an overall score. A score greater than 5 indicates poor sleep quality and less than or equal to 5, good sleep quality^21^. We applied the version validated for Portuguese-BR^22^.

**Nocturnal cramps:** the presence or of nocturnal cramps was evaluated using an evaluation form, through self-report (question: “do you have nocturnal cramps while sleeping?”, yes/no).

### Actigraphy

A non-invasive method that is widely used in the study of the sleep-wake cycle because it allows continuous monitoring in conditions closest to the individual’s routine^23^. It has an accuracy of about 80% when compared to polysomnography^24^, the gold standard for measuring sleep.

Volunteers were instructed to use the device (*Motionlogger Actigraph)* for at least 10 days on the non-dominant wrist. The data were analyzed using *Action W - Version 2* software that provided the following parameters: sleep latency, sleep efficiency, total sleep time, daytime naps (duration and time), and wake time after sleep (WASO).

During the period of actigraphy recording, the volunteers filled out sleep log and the data was analyzed together with actigraphy data for further analysis. We had three volunteers who did not fill out the diary because they were illiterate.

**General procedures:** an initial assessment form was applied to collect demographic data, the CMTNS score was applied to assess the severity of the disease, and a clinical evaluation was performed to collect anthropometric data. Then, the volunteers filled out the other questionnaires and an actigraphy was initiated.

### Statistical analysis

The final score in the questionnaires and the parameters obtained from the actigraphy were considered as dependent variables. The group (control, CMT1, and CMT2), nocturnal cramps (without or with), and gender (male or female) added as factors and age, BMI, and CMTNS score as covariates. The generalized linear model was applied, adjusting the data distribution according to the dependent variable. The normal, gamma and inverse Gaussian distributions were tested, and the best fitting distribution was chosen, according to AIC and R^2^.

The *post hoc* test adopted was that of Bonferroni and the significance level was set at 5% (*p*<0.05). A logistic regression was made to see if the group was a factor associated with the nocturnal cramps. For correlation analysis, we used Pearson’s linear correlation.

The analysis was performed using Jamovi software version 1.2.27. The effect size was calculated and interpreted according to Lenhard and Lenhard (2016)^25^.

## RESULTS

This study was carried out with 55 volunteers of which 10 presented the CMT type 1 (CMT1), 23 the CMT type 2 (CMT2), and 22 controls (CT). Table 1 shows the socio-demographic characteristics of the population.

**Table 1:** Sociodemographic characterization of the population of this study. Continuous variables are represented as mean±standard deviation and categorical variables as absolute frequency and percentage.

	CT (N = 22)	CMT1 (N = 10)	CMT2 (N = 23)
Age	34.7 ± 12.4 (range 18-61)	41.3 ± 12.6 (range 23-56)	39.5 ± 12.2 (range 19-58)
BMI	24.0 ± 3.0	26.8 ± 3.0	23.0 ± 2.9
Sex	11 women (50%) 11 men (50%)	8 women (80%) 2 men (20%)	13 women (57%) 10 men (43%)
Nocturnal cramps	16 absent (73%) 6 with (27%)	0 absent (0%) 10 with (100%)	8 absent (35%) 15 with (65%)a

a Logistic regression (*p*<0.05), OR=5.0 (95% CI: 1.4-17.8).

We have observed a significant association between nocturnal cramps and Charcot-Marie-Tooth disease type 2: CMT2 was five times more likely to have nocturnal cramps than control group (*p*=0.013, OR=5.0, 95% CI: 1.4-17.8). We did not observe a significant association for CMT1 (*p*=0.99).

### Disease severity

**Figure 1:**
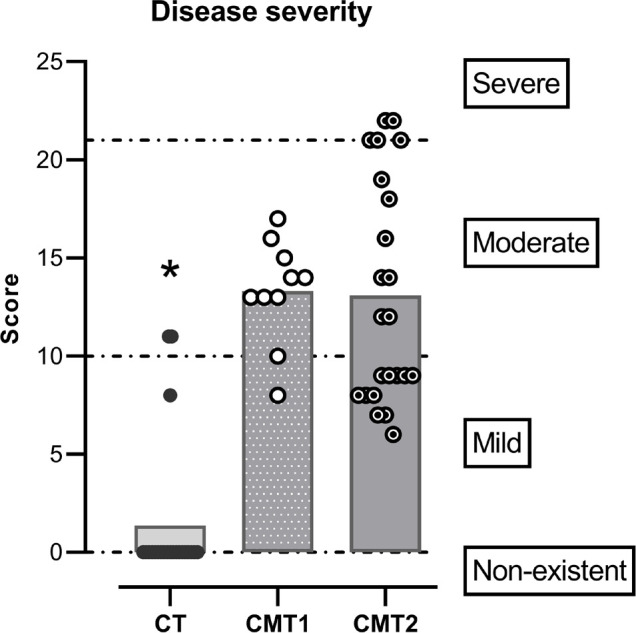
The severity of disease according to CMTNS score. Each symbol represents the individual score on the scale, the bars the means of each group, and the dotted line the cutoff points for the categories represented on the right. Notes: *Generalized linear model (p<0.05). CT presented lower values than the other groups.

In the CMT1 group, one individual (10%) presented a mild manifestation of the disease and the remaining, a moderate one (90%). No volunteer in this group showed a severe manifestation of the disease. In the CMT2 group, eleven volunteers (48%) presented a mild manifestation, seven a moderate one (30%), and five, severe (22%) (Figure 1).

For the control group, nineteen (86%) did not present any manifestation, one (5%) a mild one, and two (9%) moderate (Figure 1).

We observed a significant difference in the CMTNS score (Wald=60.2, Df=2, *p*<0.001). The difference between the means from the control group and CMT1 was 10 points and, between the CMT2 group and the control group, it was 11.2 points (*model fit* AIC=321.3, R^2^=0.73) (Figure 1).

### Pain and fatigue perception

For pain perception, we observed a significant effect of the group (Wald=7.1, Df=2, *p*=0.029). The CMT1 group had a lower score than the CMT2 group (β=-2.6, *p*=0.030), which indicates that that CMT1 has a lighter pain perception than CMT2. We did not observe significant differences between the control group and CMT1 (*p*=0.84) or CMT2 (*p*=1.0) (Table 2). We did not observe a significant effect of the disease severity (Wald=2.6, Df=1, *p*=0.11) nor of the nocturnal cramps (Wald=3.7, Df=1, *p*=0.053) (*model fit* AIC=258.1, R^2^=0.46).

**Table 2:** Results of subjective evaluations. The values are represented as mean±standard deviation (95% confidence interval).

	CT (N = 22)	CMT1 (N = 10)	CMT2 (N = 23)
Pain perception (BPI)	2.6 ± 2.3 (1.6 - 3.6)	4.1 ± 1.6 (3.1 - 5.1)	5.8 ± 3.0 a (4.6 - 7.0)
Fatigue perception (CFS)	3.1 ± 2.4 (2.0 - 4.1)	3.9 ± 2.8 (2.2 - 5.6)	6.2 ± 3.9 * (4.6 - 7.8)
Daytime sleepiness (ESS)	8.5 ± 4.5 (6.6 - 10.3)	9.2 ± 4.3 (6.5 - 11.9)	8.7 ± 4.6 (6.9 - 10.6)
Subjective sleep quality (PSQI)	7.2 ± 3.7 (5.6 - 8.8)	13.0 ± 1.9 (11.8 - 14.2)	11.7 ± 5.1 (9.6 - 13.8)

* Generalized linear model, (*p*<0.05) CMT2 higher values than the other groups;

a Generalized linear model, (*p*<0.05) CMT2 lower values than CMT1.

For fatigue, we observed a significant effect of the group (Wald=10.6, Df=2, *p*=0.005): CMT2 presented a higher score than CMT1 (β=-2.8, *p*=0.048) and control (β=-4.3, *p*=0.006), which indicates that patients with type 2 of the disease present a worse perception of fatigue than those with type 1 and controls (Table 2). We did not observe an effect of the cramps (Wald=0.6, Df=1, *p*=0.42) nor the disease severity (Wald=1.0, Df=1, *p*=0.32) (*model fit* AIC=288.7, R^2^=0.25).

### Daytime sleepiness and subjective sleep quality

For daytime sleepiness, we did not observe significant differences between groups (Wald=1.1, Df=2, *p*=0.59) (Table 2). There was also no effect of the cramps (Wald=0.3, Df=1, *p*=0.59) nor the severity of the disease (Wald=0.006, Df=1, *p*=0.94).

We also did not observe any significant effect of the group (Wald=0.2, Df=2, *p*=0.90) and the cramps (Wald=3.6, Df=1, *p*=0.057) on the subjective sleep quality (Table 2). However, we found a significant effect of the severity of the disease (Wald=7.1, Df=1, *p*=0.008). At each point of increase in the CMTNS score the chance of worsening sleep quality increases by 1.6 times (*model fit* AIC=297.5, R^2^=0.39).

We did not observe a significant linear correlation between subjective sleep quality and pain perception (*p*=0.18); however, we found a weak positive correlation between PSQI scores and fatigue (r=0.33, *p*=0.017).

### Actigraphy

The sleep parameters obtained from the actigraphy register are shown in Table 3.

**Table 3:** Actigraphy record results. The values are represented as mean±standard deviation (95% confidence interval).

	CT (N = 22)	CMT1 (N = 10)	CMT2 (N = 23)
Latency (minutes)	14.2 ± 13.9 (8.4 - 20.0)	9.9 ± 7.5 (5.2 - 14.6)	13.5 ± 6.2 (11.0 - 16.0)
Total sleep time (hours)	5.3 ± 1.2 (4.7 - 5.8)	5.5 ± 1.5 (4.6 - 6.4)	5.3 ± 1.2 (4.9 - 5.8)
WASO (minutes)	37.0 ± 26.3 (26.0 - 48.0)	35.7 ± 32.8 (15.4 - 56.0)	96.7 ± 85.7 * (61.7 - 132.0)
Efficiency (%)	90.4 ± 6.3 (87.8 - 93.0)	90.5 ± 10.4 (84.0 - 97.0)	84.4 ± 8.7 a (80.9 - 87.9)

* Generalized linear model, (*p*<0.05) CMT2 higher values than the other groups;

a Generalized linear model, (*p*<0.05) CMT2 lower values than CT group.

We did not observe significant differences between the groups for sleep latency (Wald=3.0, Df=2, *p*=0.22), but we saw an effect of cramps (Wald=7.5, Df=1, *p*=0.006) and age (Wald=23.4, Df=1, *p*<0.001) on this parameter (*model fit* AIC=358.3, R^2^=0.41). The individuals who did not have cramps presented higher latency (15.8±12.8) than those who had this complaint (11.0±7.0) (β=-5.4, *p*=0.003). Each year added to the age there is an increase of 24 seconds in sleep latency (β=0.4). No effect of the severity of the disease was observed (Wald=0.9, Df=1, *p*=0.35) (*model fit* AIC=358.3, R^2^=0.41).

For the total sleep time, no significant effect of the group (Wald=0.3, Df=2, *p*=0.85), nor the cramps (Wald=0.08, Df=1, *p*=0.46) nor the disease severity (Wald=0.08, Df=1, *p*=0.78) were observed (*model fit* AIC=187.0, R^2^=0.096).

For the wake after sleep onset (WASO), we observed a significant difference between the groups (Wald=11.5, Df=2, *p*=0.003): the CMT2 group presented WASO greater than the CMT1 group (β=-47.4, *p*=0.021) and the control group (β=-55.4, *p*=0.019). We also observed a significant effect of the cramps on this parameter (Wald=8.0, Df=1, *p*=0.005), individuals who had this complaint (71.6±80.7) spent more time awake after sleep onset than those who did not (49.5±37.5) (β=-12.1, *p*=0.041). We did not observe a significant effect of the disease severity (Wald=0.3, Df=1, *p*=0.57) (*model fit* AIC=547.3, R^2^=0.31).

For sleep efficiency, we observed a significant difference between the groups (Wald=6.7, Df=2, *p*=0.034): the CMT2 group presented lower efficiency than the control group (β=9.6, *p*=0.017). No effect of cramps (Wald=0.01, Df=1, *p*=0.91) or the severity of the disease (Wald=1.3, Df=1, *p*=0.25) was observed (*model fit* AIC=391.6, R^2^=0.19).

**Figure 2:**
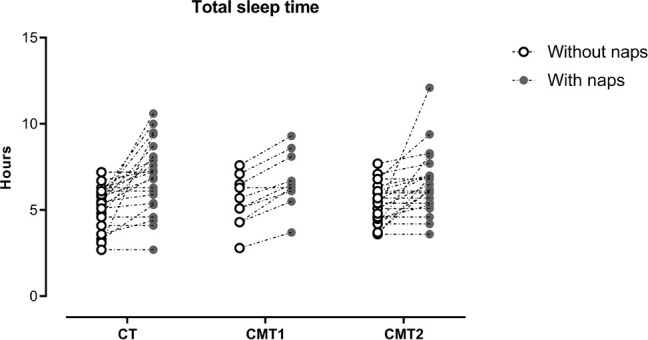
Effects of naps on total sleep time. Each symbol represents the individual values, the open symbols represent the total sleep time without the naps, and the closed ones, with the naps.

When analyzing the descriptive statistics for total sleep time, we observed that all groups presented values below the recommended for the age group^26^. Therefore, we decided to evaluate the size of the effect of daytime naps on this parameter. We found a large effect in the control group (*d*=1.0), moderate in CMT1 (*d*=0.7), and large in CMT2 (*d*=0.8) of naps on total sleep time (Figure 2).

## DISCUSSION

Our results showed that individuals from our sample affected with CMT type 2 have more nocturnal cramps, a more severe perception of pain and fatigue (Table 2), and, also, spend more time awake after the onset of sleep and have lower sleep efficiency (Table 3), which indicates higher fragmentation of sleep.

In the previous study conducted by our group, individuals with CMT type 2 showed similar results, with increased WASO, indicative of sleep fragmentation, and changes in sleep architecture^9^.

This data replicability using two different techniques for sleep assessment - actigraphy and polysomnography - is interesting because it indicates that the results are robust and that these changes are in fact characteristic of CMT disease, especially the in type 2. Neves and Kok (2011)^6^ showed that individuals from this family with this subtype of the disease presented a more severe disease manifestation, which may be related to these results.

Although polysomnography is considered the gold standard for sleep assessment, there is the effect of the first night - the bias of lack of adaptation to the environment in which polysomnography is being performed^27^ and, therefore, depending on the study, the use of actigraphy can be an alternative.

Actigraphy measures locomotor activity and has some limitations such as low specificity, which can generate spurious data^23,24^ and there are no reference values established in the literature for actigraphy^28^.

Our initial hypothesis was corroborated by our data: nocturnal cramps and pain perception appears to be related with sleep. Volunteers that had nocturnal cramps presented a more severe pain perception, reduced sleep latency, and increased WASO. The literature shows that nocturnal cramps and pain impair sleep, but different from what we observe, the sleep latency is higher^8,12,29-32^.

These conflicting data may come from the sample size or even is characteristic of the disease. The lack of previous sample calculation is a limitation. We consider that the sample calculation is an important step to delimit the power and effect size of the study, but it was not performed due to the characteristics of the disease (rare) and the procedures adopted in this study that require commitment from patients.

It is also important to point out that although we selected individuals to make up the control group without signs and symptoms of the disease, three of them scored on the CMTNS scale. Despite the absence of symptoms, the assessment of motor and sensory manifestations may have influenced our results, although we controlled for this factor in the statistical analysis.

Although pain and fatigue are not synonyms, they usually occur together: chronic fatigue is usually observed in individuals with chronic pain^33^, which may explain the similar results observed for these evaluations in our study. Also, data from the literature show that cramps can alter pain perception: the spontaneous firing of neurons that results in muscle contraction leads to the accumulation of metabolites and local ischemia, which can cause pain^34^.

As for daytime sleepiness, we did not observe differences between groups or an effect of the cramps, which may be related to the possibility that volunteers had to fit daytime naps into their routine (Table 2 and Figure 2). The motor limitations of the disease can lead to the need for sleep^35^, and since volunteers could take naps during the day, they did so. Besides, it is worth noting that there is some confusion between fatigue and daytime sleepiness^36^, which may have had an impact on volunteers’ self-reported perception.

We did not observe significant differences between the groups for the subjective sleep quality (Table 2). In a previous study conducted by our group, it was observed that 75% of the CMT2 patients had poor subjective sleep quality, which was mainly caused by the difficulty to initiate sleep, and that these changes were dependent on the severity of the^9^. In this study we also observed that a more severe manifestation of the disease led to a worse subjective sleep quality of sleep, however, the proportion of individuals with a more severe manifestation of the disease is lower, which may explain the absence of difference between groups for the subjective quality of sleep.

We did not observe significant differences between groups for total sleep time and latency. We highlight again the effects, from moderate to large, of daytime naps (Figure 2). The case and control group had total sleep time below the recommended for the age group^26^ an indication of sleep deprivation, which would be compensated in some way by daytime naps.

Since the control group presented total sleep time below the recommended (Table 3), like the case groups, and three of them scored in CMTNS evaluation (Figure 1) it is possible that the choice of individuals from the same family, had an impact on these results, which can be considered a limitation. However, this choice was important to keep social conditions as similar as possible. Future studies may use other population paired by sex, age, rather than close relatives.

## CONCLUSION

Therefore, we can conclude that sleep in Charcot-Marie-Tooth disease is impaired, specifically for those with type 2 of the disease, which is evidenced by longer awake time after sleep onset, lower sleep efficiency, and worse pain perception. We can also state that nocturnal cramps worsen sleep and pain perception in those affected by it.
